# Knowledge and attitude of healthcare workers about middle east respiratory syndrome in multispecialty hospitals of Qassim, Saudi Arabia

**DOI:** 10.1186/1471-2458-14-1281

**Published:** 2014-12-16

**Authors:** Muhammad Umair Khan, Shahjahan Shah, Akram Ahmad, Omotayo Fatokun

**Affiliations:** Department of Clinical Pharmacy, UCSI University, No. 1 Jalan Menara Gading, Taman Connaught, 56000 Cheras, Kuala Lumpur, Malaysia; Department of Pharmacy Services, Al-Qassim Hospital, Qassim, Saudi Arabia

## Abstract

**Background:**

With the increase in prevalence of Middle East Respiratory Syndrome (MERS), healthcare workers (HCWs) are at risk of acquiring and subsequently transmitting this lethal virus. In view of this, HCWs were evaluated for their knowledge of and attitude towards MERS in Saudi Arabia.

**Methods:**

A cross sectional study was performed in two hospitals of Qassim region in Saudi Arabia. A total of 280 healthcare workers were selected to participate in this study. Knowledge and attitude were assessed by using self-administered and pretested questionnaire. Descriptive statistics were carried out to express participants’ demographic information, mean knowledge score and mean attitude score of HCWs. Inferential statistics (Mann–Whitney U test and Kruskal Wallis tests, p < 0.05) were used to examine differences between study variables. Chi squares tests were used to assess the association between study variables and attitude questions. Spearman’s rho correlation was used to identify the association between the knowledge, attitude scores.

**Result:**

Participants demonstrated good knowledge and positive attitude towards MERS. The mean scores of knowledge and attitude were 9.45 ± 1.69 (based on 13 knowledge questions) and 1.82 ± 0.72 (based on 7 attitude questions). The correlation between knowledge and attitude was significant (correlation coefficient: 0.12; P <0.001). HCWs were less educated about the management (42.4%), source (66%) and consequences of MERS (67.3%), while a majority of them were well aware of the hallmark symptoms (96%), precautionary measures (96%) and hygiene issues (94%). Although the majority of respondents showed positive attitude towards the use of protective measures (1.52 ± 0.84), their attitude was negative towards their active participation in infection control program (2.03 ± 0.97). Gender and experience were significantly associated with knowledge and attitude (P < 0.05).

**Conclusions:**

The findings of this study showed that healthcare workers in Qassim region of Saudi Arabia have good knowledge and positive attitude towards MERS. Yet there are areas where low knowledge and negative attitude of HCWs was observed. However, studies are required to assess the knowledge and attitude of HCWs at national level so that effective interventions could be designed as surveillance and infection control measures are critical to global public health.

## Background

Middle East Respiratory Syndrome – Corona virus (MERS-CoV), a novel virus belonging to genus Beta coronavirus was first reported in Saudi Arabia in September 2012 [1]. Currently this virus has penetrated into countries in and near the Arabian Peninsula [2]. However, since the last update (27 March, 2014), 290 cases have been reported in the Kingdom of Saudi Arabia (KSA). It has also been reported that 20.4% of MERS victims were healthcare workers (HCWs) [3]. Initially, the eastern region of KSA was affected by MERS, however reports have revealed that the virus has penetrated throughout the country including the region of Qassim [4]. Qassim is well-known to hold world largest camel market in the world. This region is also described as having plentiful water, lots of fruit trees, palm trees and greenery. The reproduction of bats is generally very high because of such environment and it may infect camels with MERS-CoV [5]. This pose a great threat to public health as people from all over Saudi Arabia and the neighbouring countries visit this market and this could escalates the transmission of infection nationwide. The large number of reported cases from KSA also reflects the transmission of this infection in healthcare settings [6]. Another study conducted earlier in 2013 reported the transmission of the virus through a hospital cluster, suggesting the mode of spread through contact and in the form of droplets [7]. Fever with chills/rigors, cough, shortness of breath, myalgia and gastrointestinal problems (diarrhoea, vomiting, abdominal pain) are the common symptoms reported by researchers. Abnormal findings of chest radiograph are very common in MERS patients, while laboratory reports have shown thrombocytopenia, lymphopenia and increase concentration of lactate dehydrogenase and aspartate aminotransferase. The mortality rate was found to be approximately 60% [4]. It has been reported that infection control measures can keep the virus at bay. However if the required actions are not taken promptly, it may cause significant disease burden on society and results in number of needless human deaths [6]. The occurrence of asymptomatic and subclinical MERS-CoV in community or in healthcare settings could be a huge threat to public health. In view of this, HCWs are at great risk of acquiring this infection or become a source of transmission to patients and their colleagues. The presence of this fatal virus among HCWs brings into light the urgent need of developing a thorough awareness program by initiating infection control measures to cut down the rate of this rapid prevailing disease.

The Ministry of health, KSA has responded promptly to this outbreak and has designed guidelines for educating HCWs based on World Health Organization recommendations [8]. These guidelines strictly instructed the workers to follow standard precautions in interactions with patients. Therefore this study was conducted to assess the knowledge and attitude of HCWs towards MERS in Qassim region of Saudi Arabia.

## Methods

### Study design, site and participants

A cross sectional study was conducted for the period of 2 months in two multispecialty hospitals of Al-Qassim region, Saudi Arabia. These hospitals were private teaching based hospitals which serve the major proportion of Qassim population due to their multispecialty and provision of enhanced clinical services as per international standards. The Healthcare workers including Physicians, Pharmacists, Nurses and Laboratory staff were considered eligible to take part in this study. All the participants were briefed about the objectives and the outcomes of the research, those who agreed to sign the consent form were enrolled in this study. A total of 280 healthcare professionals working in the studied hospitals were selected to participate in this evaluation. This sample size was calculated on the basis of Raosoft [9] software in which the population size was kept as 1000, power as 80%, response distribution as 50%, while confidence interval and margin of error was set at 95% and 5% respectively. The generated sample size was adequately powered to estimate the process parameters. A convenience sampling approach was adopted in which the respondents were recruited on ease of accessibility.

### Study instrument

The data was collected through a self-administered questionnaire. The questionnaire was distributed to the participants by one of the authors responsible for data collection. The same author also helped the respondents with explanations when requested by the respondents. The study instrument was designed by a team of authors after a rigorous literature review [10–13]. After an initial draft of the questionnaire was designed, it was validated in 2 steps. Firstly, the study instrument was sent to researchers and professionals from pharmacy and medical background to give their expert opinion with respect to its simplicity, relativity and importance. Secondly, a pilot study was conducted by the selecting a small sample of health care professionals (n = 12) who gave their opinions on making the questionnaire simpler and shorter. Participants from all healthcare professions were selected for the pilot study. Amendments from the participants were considered and integrated into the questionnaire, while ensuring its consistency with the published literature [10–13]. After a thorough discussion, questionnaire was finalized by the authors and subsequently distributed to the participants for their response. Reliability coefficient was calculated by using SPSS v.20 and the value of Cronbach’s alpha was found to be 0.74. The data of the pilot study was not used for the final analysis.

The questionnaire was divided into 4 parts. The first part comprised of demographic information of the respondents. The second part identified the source of respondents’ MERS knowledge. The third part assessed the knowledge of healthcare workers regarding MERS in which Yes or No option was given against each set of question. The last part determined the attitude of respondents towards MERS in which their response were evaluated through 5 point Likert scale of agreement.

The study instrument assessed the knowledge of HCWs by asking questions about the nature, aetiology, symptoms, risk group, consequences, source of transmission, prevention and treatment of MERS-CoV. Knowledge scores ranged from 0-13 and cut off level of <9 were set for poor knowledge and ≥9 for good knowledge. Assessment of attitude was carried out through 7 item questions in which the responses were recorded on 5 point likert scale. A score of 1 was given to strongly agree, 2 to agree, 3 to undecided, 4 to disagree and 5 to strongly disagree. A mean score of ≤2 was considered as positive attitude while score of 3-5 was taken as negative attitude.

### Data analysis

Data was statistically analysed using SPSS version 20. Descriptive analysis was conducted and data was reported as percentage and frequency. Chi square test was applied to find the association between dependent and independent variables. P value of less than 0.05 was considered as statistically significant. Inferential statistics (Mann–Whitney U test and Kruskal Wallis tests, p < 0.05) were also used to assess the significance among study variables. These non-parametric tests (Mann–Whitney U test and Kruskal Wallis tests) were applied due to non-normal distribution of data as was evident by significant p value (p < 0.05) for both Kolmogorov-Smirnov and Shapiro-Wilks tests value. Small sample size was another criteria which supported the use of non-parametric tests. Spearman’s rank correlation coefficient (p < 0.05) was used to evaluate the association between knowledge and attitude.

### Ethical approval

The study was approved by departmental research committee, department of pharmacy, Al-Qassim hospital (Ref # M17/SAH/2014). Furthermore, written consent was obtained from the respondents prior to participation in the study.

## Result

**Figure 1:**
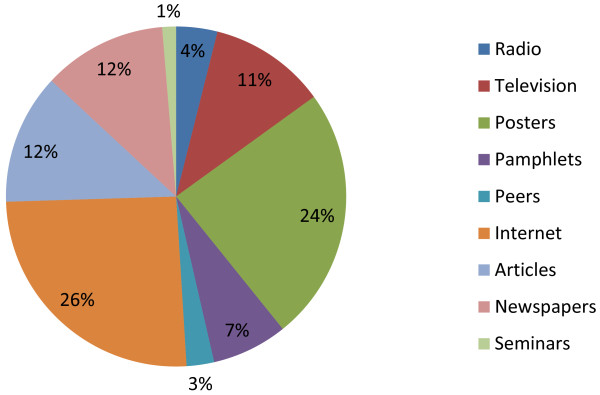
**Source of MERS information reported by HCWs.**

A total of 153 healthcare workers responded to the questionnaire giving the response rate of 54.64%. Majority of them were male (55.6%) and belonged to all major health care professions with pharmacists most in number (35.3%). The characteristics of respondents are mentioned in Table 1. The main source of MERS information reported by participants was the internet as depicted in Figure 1.

**Table 1:** **Distribution of healthcare workers according to their characteristics**

Characteristics	Healthcare workers	
	N	%
**Gender**		
Male	85	55.6
Female	68	44.4
**Age in years**		
<30	66	43.1
30-39	69	45.1
40-49	14	9.2
>50	4	2.6
**Profession**		
Physician	41	26.8
Pharmacist	54	35.3
Nurse	34	22.2
Technical Staff	24	15.7
**Years of experience**		
<3	74	48.4
3-6	36	23.5
7-10	20	13.1
>10	23	15

Table 2 describes the current status of MERS knowledge among HCWs. A total of 112 (73.2%) respondents showed good knowledge while 41 (26.8%) patients had poor knowledge of MERS. The study showed that poor knowledge was more apparent in response to questions regarding the treatment of MERS, availability of vaccines and the consequences of MERs in which the rate of incorrect responses were 57.6%, 44% and 28.8% respectively. Mean knowledge score of healthcare worker was 9.45 ± 1.69.

**Table 2:** **Knowledge of healthcare workers about MERS**

Knowledge of MERS	Correct answer	Incorrect answer
	N (%)	N (%)
MERS-CoV is caused by alpha coronavirus	118 (77.1)	35 (22.9)
MERS patients develop severe acute respiratory illness	138 (90.2)	15 (9.8)
Fever, cough and shortness of breath are hallmark symptoms of MERS	147 (96)	6 (4)
People with co-morbidity (Diabetes, cancer and other chronic diseases) are more likely to be infected	117 (76.5)	36 (23.5)
Incubation time for virus is 14-28 days	123 (80.4)	30 (19.6)
It spread through close contact with infected persons like caring and/or living	135 (88.2)	18 (11.8)
The main source of MERS virus is plant	101 (66)	52 (44)
Washing hand with soap and water for atleast 30 secs can help in prevention of transmission of disease	142 (94)	9 (6)
Vaccination of MERS virus is available in market	109 (71.2)	44 (28.8)
Polymerase Chain Reaction (PCR) can used to diagnose MERS	117 (76.5)	36 (23.5)
Special Caution must be taken when person presents with symptoms of MERS from Arabian Peninsula region	145 (96)	8 (4)
Antibiotics are first line treatment	65 (42.4)	88 (57.6)
MERS can be fatal	103 (67.3)	50 (32.7)

Note: Knowledge was assessed by giving 1 to correct answer and 0 to wrong answer. The scale measured knowledge of maximum 13 to minimum 0. Score of < 9 were taken as poor while ≥ 9 as good. Mean knowledge score was 9.45 ± 1.69.

Of 153 respondents, 126 (82.35%) showed positive attitude towards MERS while 27 (17.65%) participants displayed negative attitude about MERS. On average, the most negative attitude was shown the HCWs when asked whether their own participation in infection control program could reduce the prevalence of MERS (2.03 ± 0.97). Conversely, majority of participants responded positively when queried about the use of gowns, gloves and other protective measures when dealing with MERS patient (1.52 ± 0.84). The results are summarized in Table 3.

**Table 3:** **Attitude of healthcare workers towards MERS**

Items	Participants' responses* N (%)					p-value**			
	SA	A	U	D	SD	Prof ^1^	Gender	Age	Experience
Transmission of MERS-CoV infection can be prevented by using universal precautions given by CDC, WHO etc.^a^	42 (27.5)	90 (58.8)	9 (5.9)	12 (7.8)	0 (0)	0.006	0.002	0.016	0.250
Prevalence of MERS can be reduced by active participation of health care worker in hospital infection control program^b^	37 (24.2)	96 (62.7)	3 (2)	6 (3.9)	9 (5.9)	0.002	0.001	0.051	0.011
Any related information about MERS should be disseminated among peers and other healthcare workers^c^	57 (37.3)	78 (51)	3 (2)	3 (2)	12 (7.8)	0.006	0.001	0.448	0.071
MERS patients should be kept in isolation^d^	63 (41.2)	63 (41.2)	6 (3.9)	12 (7.8)	9 (5.9)	0.033	0.002	0.004	0.313
Intensive and emergency treatment should be given to diagnosed patients^e^	66 (43.1)	72 (47.1)	9 (5.9)	0 (0)	6 (3.9)	0.201	0.001	0.002	0.097
Healthcare workers must acknowledge themselves with all the information about MERS^f^	75 (49)	69 (45.1)	3 (2)	0 (0)	6 (3.9)	0.178	0.003	0.212	0.212
Gowns, gloves, mask and googles must be used when dealing with MERS patients^g^	90 (58.8)	57 (37.3)	0 (0)	0 (0)	6 (3.9)	0.462	0.079	0.05	0.872

^1^Profession.

*SA = Strongly agree, A = Agree, U = Undecided, D = Disagree, SD = Strongly disagree.

**derived from Chi-square test.

Note: Attitude was assessed by giving 1 to SA, 2 to A, 3 to U, 4 to D, 5 to SD. Score of < 2 were taken as positive attitude while ≥ 2 as negative attitude. Mean attitude score was 1.82 ± 0.72.

**Mean Attitude Score ± SD:** ^a^1.94 ± 0.80, ^b^2.03 ± 0.97, ^c^1.92 ± 1.08, ^d^1.96 ± 1.13, ^e^1.74 ± 0.88, ^f^1.64 ± 0.85, ^g^1.52 ± 0.84.

The association of demographic characteristics and mean knowledge and attitude questions is expressed in Table 4. Among the demographic variables, gender and experience was significantly associated with mean knowledge and attitude scores. Male participants showed more knowledge (10.1 vs 8.65, p = 0.001) and positive attitude (1.70 vs 1.93, p = 0.005) towards MERS as compared to their female counterparts. Similarly, it was also revealed that experienced personnel have more knowledge and positive attitude as compared to those who are relatively new in the field. A significant difference was found in knowledge (10.11 vs 8.89, p = 0.013) and attitude (1.70 vs 2.13, p = 0.002) of HCWs with more than 10 years of experience with those who had less than 3 years experience. The spearman correlation test revealed significant positive relationship between knowledge and attitude of healthcare workers about MERS (r = 0.12, p < 0.05).

**Table 4:** **Mean score of knowledge and attitude**

Description	N	Knowledge score (Mean ± SD)	Mean rank	P value	Attitude score (Mean ± SD)	Mean rank	P value
Age*							
<30	66	9.63 ± 1.39	80.27	0.445	1.98 ± 0.89	85.07	0.087
30-39	69	9.39 ± 1.63	72.94		1.73 ± 0.53	73.11	
40-49	14	10.07 ± 1.05	87.46		1.61 ± 0.41	69.79	
>50	4	9.0 ± 1.73	56.50		1.32 ± 0.32	36.25	
Gender**							
Male	85	10.1 ± 1.37	89.17	0.001	1.70 ± 0.88	68.14	0.005
Female	68	8.65 ± 1.26	78.51		1.93 ± 0.36	83.74	
Profession*							
Pharmacist	54	8.92 ± 1.84	80.67	0.533	1.78 ± 0.72	74.71	0.20
Physician	41	9.21 ± 1.56	68.56		1.71 ± 0.66	69.63	
Nurse	34	9.41 ± 2.07	80.79		1.72 ± 0.38	70.04	
Technical staff	24	9.62 ± 1.57	77.79		2.19 ± 0.98	93.25	
Experience*							
<3	74	8.89 ± 1.26	86.93	0.013	2.13 ± 0.73	90.43	0.002
3-6	36	9.58 ± 1.53	77.32		1.87 ± 0.79	83.9	
6-9	20	9.14 ± 1.24	60.30		1.77 ± 0.78	73.60	
≥10	23	10.11 ± 1.67	89.17		1.7 ± 0.41	68.62	

*Kruskal Wallis Test (p < 0.05).

**Mann Whitney Test (p < 0.05).

## Discussion

To the best of our knowledge, there are no previous reports of similar studies, particularly none that examined the healthcare workers’ knowledge and attitude towards MERS. In view of this, the comparison of our findings has been made with other related conditions like Severe Acute Respiratory Syndrome (SARS).

The findings of this study showed good knowledge and positive attitude of HCWs towards MERS-CoV. Majority of the respondents had gained knowledge about MERS from internet as shown by this study. This result is however is not supported by study which showed that participants’ main source of knowledge about such kind of virus was Television [14]. This difference could be possibly explained by the fact that the referenced study was conducted in 2004, since then a lot of advancement has been made as now healthcare workers are more reliant on internet technologies to gain knowledge on emerging disease like MERS [15]. Speculation could also be made that most of the educational materials on MERS are posted online by ministry of health which may have urged the HCWs to use internet technology to gain access to those documents [3]. However, caution must be taken when using internet to gain healthcare knowledge because of the information overload. It is difficult to determine authenticity of the source and one can easily me misguided. Therefore, emphasis should be made on the development of evaluation skill among healthcare professionals for the extraction of knowledge from internet. The ministry of health website should also be kept updated regularly and healthcare professionals must be encouraged to visit official website to seek knowledge on health related issues. Additionally, under-utilized sources like seminars and availability of research articles could also be employed in a campaign to educate HCWs regarding MERS.

Furthermore, the most number of correct responses were gathered from the question about the symptoms of MERS followed by the question on the maintenance of hand hygiene in the prevention of disease transmission. These findings may be due to emphasis by the health authorities on such issues in their awareness program. These results are in line with the findings of other studies which showed the positive response of HCWs towards hand hygiene when dealing with SARS-CoV [16, 17]. These results were very encouraging as it is known that the lack of hygiene maintenance could lead to increase morbidity and mortality of deadliest virus like MERS [14]. However, with regards to knowledge of symptoms, the result of current research is not in accordance with a study conducted in US to determine HCWs knowledge of SARS in which a poor knowledge was exhibited by respondents when asked about symptoms of SARS [18]. The discrepancy in these results could be explained by a fact that educational campaigns by relevant authorities in Saudi Arabia have focussed more on sign and symptoms of MERS which may have enhanced their knowledge in this area of MERS [19]. Another speculation is that the outbreak of MERS in Saudi Arabia is very recent and there are more talks about it among the healthcare workers and in the community. Since, the focus is more towards symptoms and prevention; this may have increased their knowledge about the disease in these areas.

Conversely, the healthcare workers were least knowledgeable regarding the management of MERS as 57.6% of workers replied wrongly when asked whether antibiotics are first choice drugs. This outcome is somewhat similar to another study in which 40% of respondents gave incorrect answer when asked about the management issue [20]. This again highlights the possibility that respondents were not thoroughly briefed about the management issues by the relevant authorities during their educational campaign and it was observed that majority of the correct answerers were given by experienced respondents. This argument is also supported by another false answer from almost 30% of the workers about the availability of vaccine. This question was again correctly answered by experienced workers in comparison to less experienced ones.

It is also noteworthy to mention the lack of respondents’ knowledge about the source of MERS-CoV. About 44% of the HCWs answered it incorrectly. Although, research has revealed that camel [21] could be the main source of MERS, and human themselves could act as a source of transmission of this disease [22], the knowledge of HCWs regarding this question was below par. It is therefore necessary to uncover this aspect of MERS so that HCWs can play their part by educating people to counter the threat of MERS to global public health.

The mean attitude score was found to be in the positive range. The most positive attitude of healthcare workers was regarding the use of protective equipment when dealing with MERS patient (1.52 ± 0.84). This finding is in line with another study which showed positive response from the healthcare workers that goggles and gloves should be worn when dealing with healthcare associated infections [23]. It was also observed that age was significantly associated with this question as the aged people (≥40 years) more positively respond to this question as compared to younger ones (<40 years). On the contrary, the only negative attitude was observed when respondents adversely replied to the question of whether their active participation in infection control program can reduce the prevalence of MERS. Profession, gender and experience were found to be significantly associated with this question. Physicians, male and more experienced workers (≥7years) showed relatively more positive attitude in reply to this question. This result is not in accordance with another study in which the highest attitude of healthcare workers was noted towards active participation in hospital control program [24]. The effect of gender and attitude could be well explained by the traditional norms and customs in Saudi Arabia. Males have more interaction and socialization than females. Their inclination towards meeting other healthcare professionals and specialists is more as compared to their female counterpart. This could influence their attitude towards their involvement in infection control measures to reduce the prevalence of MERS. Similarly, physicians were more positive in attitude as compared to healthcare professionals because of their in depth clinical training on infection control and greater opportunities of professional development as compared to other team members. The results are in agreement with other studies which have reported positive attitude of physicians among all healthcare workers [25, 26]. There is a need to improve the adherence of all HCWs to universal precautions of hospital infection as suggested by other investigators [27].

Overall, gender and experience were the two demographic variables significantly associated with the mean knowledge and attitude scores. Although the relationship of experience with knowledge and attitude has been reported significant by studies [28], other research does not support the association of gender with the knowledge and attitude of healthcare workers [29]. This variation could be possibly explained by the traditional and cultural norms of Saudi Arabia in which males are more exposed to healthcare system as compared to females. The constitutional and legal system has sanctioned more superiority to male in terms of interaction of with other professionals, traveling around the world for symposiums, conferences and other health related activities. All these issues may affect the knowledge and attitude of female healthcare workers [30, 31].

The positive correlation between knowledge and attitude of healthcare workers reaffirms the association between knowledge and attitude with MERS. In view of this, it could be established that HCWs with more positive attitude towards MERS are motivated to seek more information and develop their knowledgebase around the disease. The reason of this correlation could be explained by the theory of Reasoned Action. A person’s intention to a specific behaviour is a function of their attitude towards that behaviour [32]. However, future studies would be required to develop an understanding of what underlies both the patterns of knowledge and the expressed attitudes of the HCWs.

The strength of this study is that it addresses a major health problem that confronts HCWs in Saudi Arabia. It has highlighted the area where very little research has been done. The findings of this study would be critical to design effective control measures of MERS in an outbreak situation. The use of a 2 step approach to validate the questionnaire tool was another strength of this study as it enhances the confidence in the findings that result from this tool. Despite of the study findings, we acknowledge its limitation. The low response rate, potential sample clustering and statistical errors due to multiple significance testing may limit the generalizability of the results.

## Conclusion

HCWs in Qassim region of Saudi Arabia showed positive attitude and good knowledge of MERS; however there is still room for improvement in certain areas like the possible sources of virus transmission and the management of MERS. Extensive health education campaigns should be provided to HCWS to bridge the gap between the current and the required knowledge by focussing on less knowledgeable areas. The study recommends establishing professional and occupational campaigns to augment the knowledge of HCWs which would also positively influence their attitude towards MERS.
